# Comparative Study on the Stiffness of Poly(lactic acid) Reinforced with Untreated and Bleached Hemp Fibers

**DOI:** 10.3390/polym15132960

**Published:** 2023-07-06

**Authors:** Roberto J. Aguado, Gabriela A. Bastida, Francisco X. Espinach, Joan Llorens, Quim Tarrés, Marc Delgado-Aguilar, Pere Mutjé

**Affiliations:** 1 LEPAMAP-PRODIS Research Group, University of Girona, C/Maria Aurèlia Capmany 61, 17003 Girona, Spain; 2 Instituto de Tecnología Celulósica, FIQ-CONICET, Universidad Nacional del Litoral, Santiago del Estero 2654, Santa Fe S3000AOJ, Argentina; 3 CATS Research Group, Department of Architecture and Construction Engineering, University of Girona, Avda Mª Aurelia Capmany 61, 17071 Girona, Spain

**Keywords:** biocomposites, cellulose fiber, micromechanics, natural fibers, poly(lactic acid), Young’s modulus

## Abstract

Composite materials containing natural reinforcement fibers, generally called biocomposites, have attracted the interest of both researchers and manufacturers, but the most environmentally advantageous combinations include a bio-based matrix, as well. With this in mind, a poly(lactic acid) (PLA) matrix was reinforced with natural fibers from hemp, both untreated strands (UHSs) and soda-bleached fibers (SBHFs). The preparation of the subsequent fully bio-sourced, discontinuously reinforced composites involved kinetic mixing, intensive single-screw extrusion, milling, and injection molding. Up to a fiber content of 30 wt%, the tensile modulus increased linearly with the volume fraction of the dispersed phase. Differences between SBHFs (up to 7.6 Gpa) and UHSs (up to 6.9 Gpa) were hardly significant (*p* = 0.1), but SBHF-reinforced composites displayed higher strain at failure. In any case, for the same fiber load (30 wt%), the Young’s modulus of PLA/hemp biocomposites was greater than that of glass fiber (GF)-reinforced polypropylene (5.7 GPa), albeit lower than that of PLA/GF (9.8 GPa). Considering all the measurements, the contribution of each phase was analyzed by applying the Hirsch model and the Tsai-Pagano model. As a concluding remark, although the intrinsic tensile modulus of SBHFs was lower than that of GF, the efficiency of those natural fibers as reinforcement (according to the rule of mixtures) was found to be higher.

## 1. Introduction

Bioplastic/natural fiber composites can be thought as the intersection between two sets of materials. On one side stand bioplastic composites, in which the reinforcement phase may consist of wood fiber, wood flour, glass fiber (GF), or carbon fiber, among other possibilities. They may be biodegradable or non-biodegradable. Their market size was valued at USD 30.9 billion in 2021, and is expected to grow at a compound annual growth rate (CAGR) of 10.4% [1]. On the other side, we can find the so-called biocomposites, which are reinforced with wood or other natural fibers, but their matrix may (or may not) be synthetic. Their CAGR has been projected to be as high as 16% [2]. Thence, it follows that prospects for bioplastic/natural fibers composites share some of the optimism of these two sets in which they participate.

Regarding the bioplastic matrix, one of the most popular choices is poly(lactic acid) (PLA), due to a number of reasons that have been discussed in depth elsewhere [3,4]. One of them is its high stiffness, which can be even further enhanced, as is known for other thermoplastic matrices, by fiber reinforcements. Indeed, the tensile modulus of polypropylene (PP), polycarbonate and different polyamides, for example, can be more than doubled with the incorporation of glass fiber (GF) [5,6,7]. Hence, GF-reinforced composites are usually chosen in the so-called “stiffness-limited design”. This term refers to the design of columns, panels, shafts, building blocks, or any other component or product that prioritizes avoiding or minimizing elastic deformation [8].

Despite the environmental advantages of natural fibers, mainly in terms of energy consumption and carbon footprint [9,10,11], their market share still goes far behind GF in the current composites market [12]. Determining the optimal process to incorporate natural fibers, such as those of hemp, flax, or jute, as a discontinuous reinforcement phase into thermoplastic matrices remains a pending issue. Various compatibilizers and chemical modifications have been suggested to promote dispersion [13]. Strategies for improving dispersion and/or interfacial compatibility often involve either reactions on the surface of the fibers [14,15] or the use of maleic anhydride-grafted thermoplastics [16,17]. Nonetheless, this practice does not comply with the principles of green chemistry [18]. Well-known and relatively clean processes, such as soda pulping and total chlorine bleaching, have been shown to enhance the tensile strength of PLA/hemp composites [19], but at the expense of significant material losses.

Strands and fabrics from hemp, both untreated and after undergoing different chemical processes, have already been incorporated into PLA matrices [15,20,21]. Song et al.’s [20] biocomposites [3,20], comprising PLA and degummed hemp strands (cooking in alkali, 100 °C), reached tensile strength improvements of up to 39% over the initial bioplastic. We hypothesize that bleached soda-anthraquinone hemp fibers (BSHFs) with a high content of fines would attain better interaction after extensive blending processes. This is expected from the fact that they possess even less lignin and more surface area for intermolecular interactions [22].

This work analyzes the stiffness of PLA composites reinforced with untreated hemp strands (UHSs) and with soda-bleached hemp fibers (SBHFs). In both cases, the reinforcement phase was discontinuous, and the same methods of compounding and molding were implied. The research question addressed in this work was “Does the biocomposite benefit from delignification and subsequent disintegration of the middle lamellae of natural fibers?” Compatibilizers, binders, and derivatizing agents were avoided. We describe the production of specimens for tensile tests using kinetic mixing, extrusion (twice), and injection molding. Then, the stiffness of PLA/SBHF materials is compared to that of PLA/UHS and unreinforced PLA. To draw conclusions on their potential applications, the fully green composites proposed here are also compared to conventional GF-reinforced PP.

## 2. Materials and Methods

### 2.1. Materials

The PLA matrix of the biocomposites described here was Ingeo™ Biopolymer 3251D from NatureWorks (Plymouth, MN, USA). Agrofibra S.L. (Puigreig, Spain) kindly provided untreated hemp strands (UHSs). Total chlorine-free bleached soda pulp from hemp strands, with an ISO brightness value of 89.5%, was supplied by Celesa (Tortosa, Spain).

Basic chemical characterizations of both UHSs and SBHFs were carried out according to TAPPI standards for lignocellulosic materials (T 204, T 211, T 249, T 429, T 222, UM 250) [23]. Additionally, their crystallinity index was estimated by applying Segal’s method [24] on X-ray diffraction patterns attained by means of an X’Pert MPD X-ray diffractometer from Philips (Philips Ibérica, Valencia, Spain) with auto-divergent slits and Cu-Kα radiation (45 kV, 40 mA).

### 2.2. Preparation of Biocomposites

**Figure 1:**
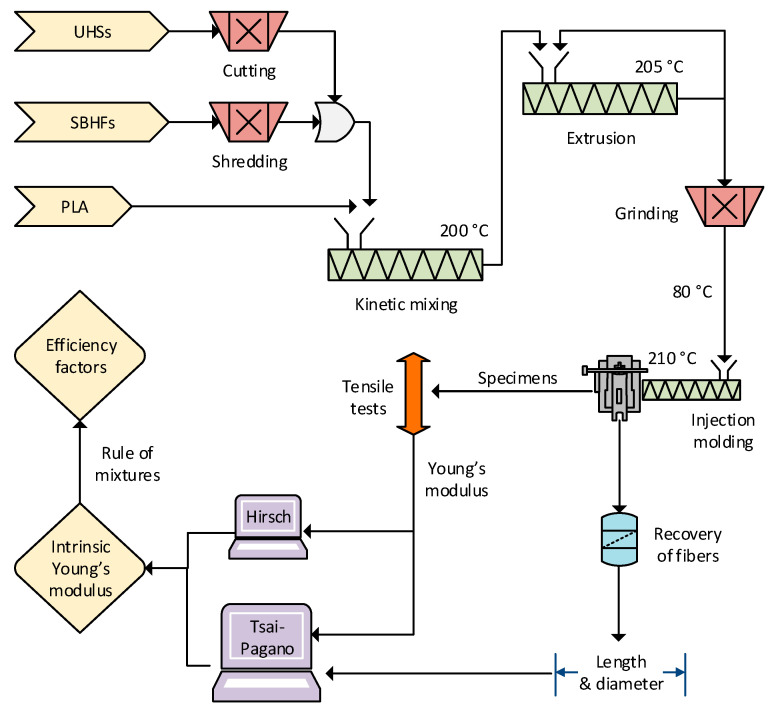
Simplified diagram of the experimental procedure and the calculation methodology.

To produce the composites, PLA was mixed with 10 wt%, 20 wt%, and 30 wt% of either UHSs or BSHFs. The procedure is summarily schematized in Figure 1. For combinations, UHSs were cut down to lengths of 3.0 ± 0.3 mm, while SBHF dry pulp boards underwent fractionation using a paper shredder. In both cases, fibers were dispersed in the matrix using a Gelimat™ G5S kinetic mixer (Dusatec, Ramsey, NJ, USA). We set the angular speed at 2500 rpm, the discharge temperature at 200 °C, and the mixing time at 3 min. Then, the combined material was passed twice through a single-screw extruder, Eurotecno 3035 D (Eurotecno, Sant Fost de Camcentelles, Spain), with the screw rotating at 40 rpm. The temperature increased from 180 °C (hopper) to 205 °C (die). Finally, the extrudate was granulated in a hammer mill and stored at 80 °C for 24 h.

We obtained dog-bone specimens (type I, ASTM D790) for tensile tests by means of an injection molding machine from Arburg (Lossburg, Germany), 220 M 350-90U [25]. The processing temperature increased from 170 °C (hopper) to 210 °C (nozzle). The injection pressure ranged from 50 MPa to 80 MPa, depending on the fiber load.

### 2.3. Characterization of Biocomposites

Specimens were conditioned under standard conditions of temperature and humidity [26]. After 24 h, we performed tensile tests at 2 mm/min on up to ten samples, according to ASTM D3039 [25]. The tests were performed using a Universal Testing Machine, Instron 1122 (Barcelona, Spain), equipped with a 5 kN load cell and an extensometer. Additional experiments were performed with UHSs, following the ASTM standard D3379-75(1989)e1 for high-modulus single-filament materials [27].

The reinforcement fibers were recovered from the composites by the dissolution of the matrix in dichloromethane. Recovered fibers were suspended in water and had their dimensions measured using a MorFi Compact analyzer from Techpap (Gières, France), equipped with the software MorFi v9.2.

### 2.4. Modeling and Calculation

There are many empirical or semi-empirical models used to predict the tensile properties of fiber-reinforced composites, commonly based on the rule of mixtures [28]. Indeed, the Young’s modulus of composites (*E_t_^C^*) often follows a linear trend with the volume fraction of fibers (*V^F^*), at least until a certain level of fiber load [16,29]:

*E_t_^C^* = *η_e_* × *E_t_^F^* × *V^F^* + (*1* − *V^F^*) × *E_t_^m^* (1)

where *E_t_^m^* is the modulus of the matrix, *E_t_^F^* is the intrinsic Young’s modulus of fibers, and *η_e_* is an efficiency factor, which can be expressed as the product of an orientation efficiency factor (*η_o_*) and a length efficiency factor (*η_l_*). The latter can be estimated from the Cox–Krenchel model [30,31]. In general, the higher the aspect ratio of fibers, the higher the length efficiency factor. At the same time, the product *η_e_ × E_t_^F^* is the contribution of the reinforcement and is used to obtain a fiber tensile modulus factor (*FTMF*), which corresponds to the slope of *E_t_^C^* − *(1 − V^F^) × E_t_^m^* against *V^F^*. *E_t_^F^* can be estimated from the Hirsch model [32]:

*E_t_^C^* = *β [E_t_^F^* × *V^F^* + *E_t_^m^* × *(1* − *V^F^)]* + *(1* − *β)* × *E_t_^F^* × *E_t_^m^/[E_t_^m^* × *V^F^* + *E_t_^F^ (1* − *V^F^)]* (2)

In Equation (2), the parameter *β* accounts for the capability of matrix-to-fiber stress transfer. A value of 0.4 usually yields satisfactory predictions [33,34]. Whereas this approach does not consider fiber morphology, the length (*l^F^*) and diameter (*d^F^*) of the recovered fibers are input variables for the Halpin and Tsai equations with the Tsai and Pagano solution [35,36]:

(3) $$E_{t}^{C}=\frac{3}{8}\frac{1+2\frac{l^{F}}{d^{F}}\left(\frac{\frac{E_{t}^{F}}{E_{t}^{m}}-1}{\frac{E_{t}^{F}}{E_{t}^{m}}+2\frac{l^{F}}{d^{F}}}\right)V^{F}}{1-\left(\frac{\frac{E_{t}^{F}}{E_{t}^{m}}-1}{\frac{E_{t}^{F}}{E_{t}^{m}}+2\frac{l^{F}}{d^{F}}}\right)V^{F}}E_{t}^{m}+\frac{5}{8}\frac{1+2\left(\frac{\frac{E_{t}^{F}}{E_{t}^{m}}-1}{\frac{E_{t}^{F}}{E_{t}^{m}}+2}\right)V^{F}}{1-\left(\frac{\frac{E_{t}^{F}}{E_{t}^{m}}-1}{\frac{E_{t}^{F}}{E_{t}^{m}}+2}\right)V^{F}}E_{t}^{m}$$

## 3. Results and Discussion

### 3.1. Stiffness of PLA/UHS and PLA/SBHF

**Figure 2:**
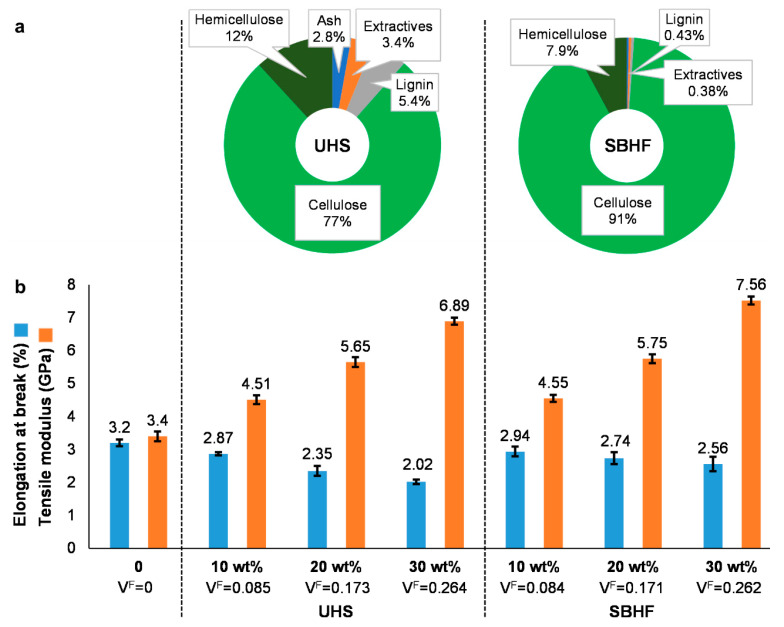
Experimental data (**a**) of the composition analysis and (**b**) of the tensile tests, encompassing Young’s modulus and strain at break (εt^C^), of PLA/UHS and PLA/SBHF biocomposites.

Soda pulping processes are known to be rather unselective, but the subsequent bleaching stage allowed for a more selective removal of lignin [37]. As shown in Figure 2a, the fractions of lignin and extractives were diminished by one order of magnitude. The density of the reinforcement phase was slightly decreased, from 1.50 g/cm^3^ to 1.48 g/cm^3^. This difference was taken into account for the calculation of the volume fraction, but it is probably negligible in terms of the contribution to stiffness.

UHSs and SBHFs approximately doubled the Young’s modulus of PLA, but the difference between both kinds of fibers was hardly significant (Figure 2b). A one-way ANOVA test at a 95% confidence rate did not allow the null hypothesis (*p* = 0.10) to be discarded. However, these similar enhancements of stiffness came along with unequal effects on the strain at failure, since PLA/SBHF composites were consistently able to withstand more plastic deformation than PLA/UHS. The strain at failure for PLA/UHS (30%) was identical to that found by Zouari et al. [38]. In all cases, the enhancement of stiffness came at the expense of lower ductility, but this detriment was less prejudicial for the composite material in the case of the fibers that underwent chemical pulping and bleaching.

On one hand, the removal of amorphous components (lignin, some hemicellulose macromolecules, and extractives) is generally expected to impart greater intrinsic stiffness [39]. On the other, given that the original strands were already rich in cellulose (77%), the extent of the enhancement attained by delignification was rather low. Also in this context, the crystallinity index of SBHFs was only slightly higher than that of UHSs (0.87 and 0.79, respectively). It is known that the degree of supramolecular order exerts a significant effect on the tensile properties of cellulosic materials [40], but that of hemp strands was initially elevated. Furthermore, the possible gains attained by removing amorphous components were plausibly hampered by the depolymerization of cellulose during alkaline pulping and bleaching [41]. In the same sense, these treatments damaged the surface of fiber hemps to the point of generating a high amount of fines (47%), which may contribute to the tensile strength of the material [19,42], but made no significant contribution to stiffness.

In a previous work with jute [43], whose initial lignin content was as high as 13%, the Young’s modulus significantly increased by decreasing the lignin content to roughly 8%, but further delignifying had negligible effects on stiffness. In the case of hemp, whose lignin content without chemical treatments lies below that amount (as long as the hemp core is removed), the importance of delignification is arguable. Furthermore, a major drawback of chemical pulping is the low material efficiency. In fact, the total yield of pulping and bleaching was 45%, meaning that 55% of the initial hemp weight was solubilized. The material that was lost this way included not only lignin (11% of the solubilized fraction), ashes (6%) and extractives (7%), but also hemicellulose (17%) and hydrolyzed cellulose (59%). Hence, the loss of cellulose was as high as 32% over the initial weight of hemp, which puts the convenience of pulping and bleaching for stiffness-limited design under question. Nonetheless, they have been proven to be of utmost relevance to enhance the tensile strength, for which the quality of the interphase has a significant influence [19].

### 3.2. Empirical Assessment of the Constituents

**Figure 3:**
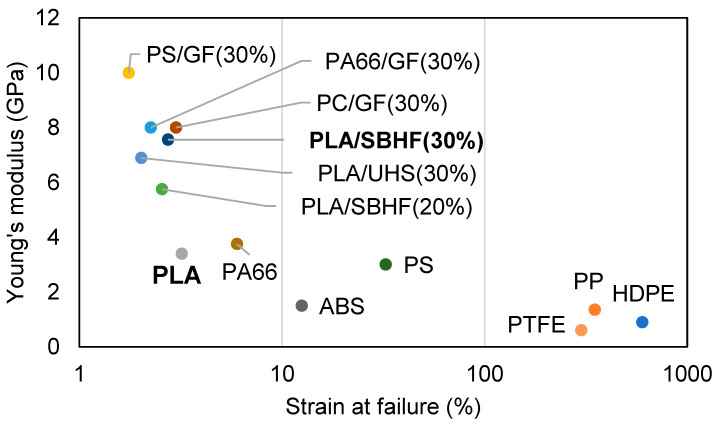
Typical stiffness and ductility of some of the most popular thermoplastic materials and their composites [44,45,46], including the biocomposites presented in this work. PS: polystyrene. PC: polycarbonate. PA66: polyamide 6,6. PTFE: polytetrafluoroethylene.

As a matrix, PLA is deemed a relatively stiff thermoplastic material, with a Young’s modulus of 3.4 GPa. For comparison purposes, it could be mentioned that the moduli of PP, high-density polyethylene (HDPE), polybutylene, poly(vinyl chloride), and acrylonitrile butadiene styrene (ABS) lie around 1.5 GPa, 0.8 GPa, 0.4 GPa, 2.8 GPa, and 2.3 GPa, respectively [44]. Figure 3 locates PLA and PLA/hemp composites in comparison with popular thermoplastic materials and GF-reinforced composites in which they constitute the matrix [45,46]. It should be noted that the indicative values displayed may belong to a broad range of possible values. In any case, the region at the right (high strain at failure) includes plausible choices for ductility design, while the upper region (high modulus of elasticity) includes materials for stiffness design.

Regarding reinforcement fibers, it is unusual for those of natural origin to match GF (much less carbon fiber). Hence, with a choice of materials depending on a stiffness-limited design in mind, natural fibers should reinforce an already stiff matrix. Tensile tests on single UHSs revealed a Young’s modulus of 25 ± 9 GPa (95% confidence). The uncertainty of the measurement arises from the heterogeneity of the material and from the limitations of the method. It should be noted that these experiments were performed with UHSs, following the ASTM standard D3379-75(1989)e1 (withdrawn). Moreover, this kind of direct measurements could not be applied to SBHFs, whose length is generally <1 mm. For these reasons, the contribution of reinforcement fibers to stiffness is better estimated using micromechanical models, such as those of Hirsch (Equation (2)) or Tsai-Pagano (Equation (3)). Commonly, values estimated from these models are higher than experimental results [34], but also more reliable when it comes to predict the mechanical properties of similar composites.

### 3.3. Micromechanics of the Tensile Modulus

Table 1 presents the average length (weighted in length), the mean diameter, and the intrinsic tensile modulus and reinforcement efficiency factors of PLA/SBHF composites. Since their *E_t_^C^* did not differ significantly from that of PLA/UHS, the latter’s micromechanical parameters are qualitatively similar.

While Equation (3) considers the influence of the aspect ratio, the intrinsic Young’s moduli, as calculated by Equation (2), differed by less than 10% from the former. Hence, with fewer inputs, the Hirsch model provided a satisfactory estimation. This estimation of *E_t_^F^* can be extrapolated to predict the contribution of SBHFs in other composite materials, i.e., in matrices other than PLA. For instance, using a value of 30 GPa for *E_t_^F^* and a value of 0.5 for *η_e_* would predict an increase in the Young’s modulus of bleached hemp-reinforced polyamides, applying the rule of mixtures (Equation (1)), from 3.4 to 7.0 GPa. In a previous work, the axial tensile modulus of said composite was measured as 6.7 ± 0.2 GPa [47]. In other words, the prediction of the Young’s modulus by the Hirsch model yielded an overestimation of only 5% with respect to the experimental results, or of 1.5–0.77% taking into account the lower and higher limits of the tolerance interval.

The mean efficiency factor was found to be 0.530 according to the Hirsch model, or 0.561 with the Tsai–Pagano model. The intrinsic Young’s modulus showed a mean value of 32.5 GPa (Hirsch) or 30.7 GPa (Tsai-Pagano). The product of both parameters, *FTMF*, was 17.4 GPa, and it quantifies the contribution of the reinforcement phase. As expected, these intrinsic Young’s moduli, slightly over 30 GPa according to both models, were lower than that of GF, 71.6 GPa [33]. They lie within the range reported in the literature for hemp fibers, roughly between 17 and 45 GPa [48]. However, hemp strands, either untreated or after a delignifying treatment, were more efficient as a reinforcement phase than GF (*η_e_* = 0.243).

As mentioned above, the efficiency factor can be expressed as a product of two contributions; that of the fiber length (Cox-Krenchel), and that of orientation. Table 2 shows their values estimated for each level of fiber load. From the mean orientation efficiency factor, 0.649 (if *E_t_^F^* is calculated from the Tsai-Pagano model), the average angle of reinforcement fibers with the axis can be estimated as 43.2°. In the hypothetical case of fibers that were perfectly aligned in the axial direction, the orientation efficiency factor would be 1. The accomplishment of such a hypothesis is highly unlikely with injection molding, especially if using a discontinuous reinforcement phase, but composites with well-aligned mats have been reported to be produced by stacking and hot pressing [49]. If this axial orientation were attained with SBHFs, reaching η_o_ values close to 1, the modified rule of mixtures would predict Young’s moduli as high as 9.4 GPa.

### 3.4. Comparison of PLA/Hemp and More Conventional Options

**Figure 4:**
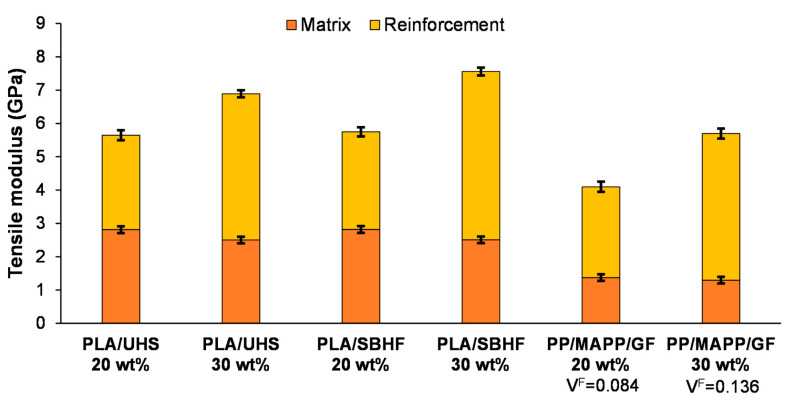
Comparison between PLA/SBHF and PP/GF in terms of stiffness, highlighting the contribution of the matrix and that of the reinforcement fibers.

Figure 4 shows the contributions of the composite constituents, namely matrix and reinforcement fibers, to the Young’s modulus of PLA/UHS, PLA/SBHF, and PP/GF with maleic anhydride–grafted polypropylene (MAPP). Two levels of fiber load were selected for this comparison: 20 wt% and 30 wt%. Overall, due to the higher stiffness of the matrix (PLA vs. PP) [50,51], the tensile modulus of PLA/hemp was significantly higher than that of a more conventional PP/GF composite.

As a drawback, PLA/hemp composites (minimum *ε_t_^C^* = 2.0%) were more brittle than PP/GF (minimum *ε_t_^C^* = 4.4%). Additionally, PLA/UHS composites were shown to withstand less tensile load (roughly 56 MPa) than PP/GF, as delignification was required to match the tensile strength of the latter (78 MPa for the same fiber load) [19]. Unlike stiffness, these two properties, i.e., ductility and tensile strength, were enhanced by alkaline pulping and bleaching, possibly due to the improvement of the fiber/matrix interfacial compatibility [52], which does not exert a capital effect when it comes to the Young’s modulus. Moreover, compatibility reasons aside, delignification allowed fibers to withstand more plastic deformation, since cellulose–lignin interfaces are zones of preferential fracture, as discovered in molecular dynamics studies [53].

An inescapable question is how PLA/hemp composites compare to PLA/GF composites; same matrix, stiffer reinforcement fibers. The latter kind of material should not be included within the umbrella term of biocomposites, given the energy-intensive manufacturing process of GF, and that it hampers the biodegradation of PLA. In fact, commercially available composites of this nature have recently been disappearing from the catalogue of some major manufacturers [54]. That said, the extraordinary stiffness of PLA/GF composites is undoubted. With a fiber load of 30 wt%, their tensile modulus has been found to be 9.8 GPa [55]. Interpolating for 20 wt%, the expected value is 7.7 GPa, which stands close to the stiffness found for Wang et al.’s silanized GF (20 wt%)-reinforced PLA (7.8 GPa) [56]. Nonetheless, although the intrinsic Young’s modulus of GF more than doubles that of hemp fibers [33], the increase in the composite’s stiffness that SBHFs impart is approximately 70% of the enhancement provided by GF. This means that the efficiency of SBHFs as a reinforcement for PLA is higher than that of GF.

All notions considered, PLA/UHS or PLA/SBHF composites are solid candidates for nearly the whole spectrum of applications of GF-reinforced thermoplastics with a stiffness-focused design. Nonetheless, the brittleness of PLA-based composites may limit their applications in aerospace parts, home appliances, or any other material meant to withstand plastic deformation before breaking down.

## 4. Conclusions

UHSs and SBHFs of up to 30 wt% (i.e., a volume fraction of 0.26) were dispersed in a PLA matrix, yielding fully bio-sourced composites with enhanced stiffness. PLA/hemp composites were found to be stiffer than some conventional reinforced materials, namely, PP/GF, although less stiff than, e.g., PLA/GF or PA66/GF. Remarkably, PLA/UHS biocomposites were more brittle (εt^C^ as low as 2.0%) than PLA/SBHF, but both were significantly more brittle than PP/GF. With tensile moduli around 7 GPa for both PLA/UHS and PLA/SBHF, the loss of material experienced during pulping and bleaching (55%) made these processes unworthy of consideration in terms of stiffness-limited design. The values estimated for the intrinsic Young’s modulus (around 30 GPa) and the efficiency factor (roughly 0.5) can be used to predict the contribution of hemp strands to other thermoplastic matrices.

## Figures and Tables

**Table 1:** Intrinsic Young’s modulus of SBHFs and efficiency, as calculated from the Hirsch [32] and the Tsai-Pagano models [35].

Load (wt%)	V^F^	l^F^ (µm)	d^F^ (µm)	Hirsch Model		Tsai-Pagano Model	
				E_t_^F^ (GPa)	η_e_	E_t_^F^ (GPa)	η_e_
10	0.085	353	20.5	32.4	0.522	31.2	0.544
20	0.171	318	20.5	32.1	0.524	30.3	0.563
30	0.262	295	20.4	33.0	0.542	30.5	0.578

**Table 2:** Breakdown of the efficiency factor in length and orientation, and average orientation angle of reinforcement fibers.

Load (wt%)	Hirsch Model			Tsai-Pagano Model		
	η_l_	η_o_	α (°)	η_l_	η_o_	α (°)
10	0.848	0.616	45.9	0.853	0.650	43.2
20	0.861	0.609	46.5	0.865	0.642	43.7
30	0.874	0.621	45.5	0.877	0.655	42.7

## Data Availability

All data are explicit in the manuscript or else available on request.
